# Supplementary material for: LC-MS/MS-Based Serum Protein Profiling for Identification of Candidate Biomarkers in Pakistani Rheumatoid Arthritis Patients
Source: Life (Basel). 2022 Mar 21;12(3):464. doi: 10.3390/life12030464 (PMC8955720; doi:10.3390/life12030464)
Supplement: Supplementary file 1 [file life-12-00464-s001.zip › life-1612791-Supplementary material.pdf]

life

MDPI

*Article*

# LC-MS/MS-based Serum Protein Profiling for Identification of Candidate Biomarkers in Pakistani Rheumatoid Arthritis patients

**Sidrah Jahangir [1], Peter John [1,*], Attya Bhatti [1], Muhammad Muaaz Aslam [2], Javaid Mehmood Malik [3], James R Anderson [4] and Mandy J Peffers [4]**

**Supplementary Materials:**

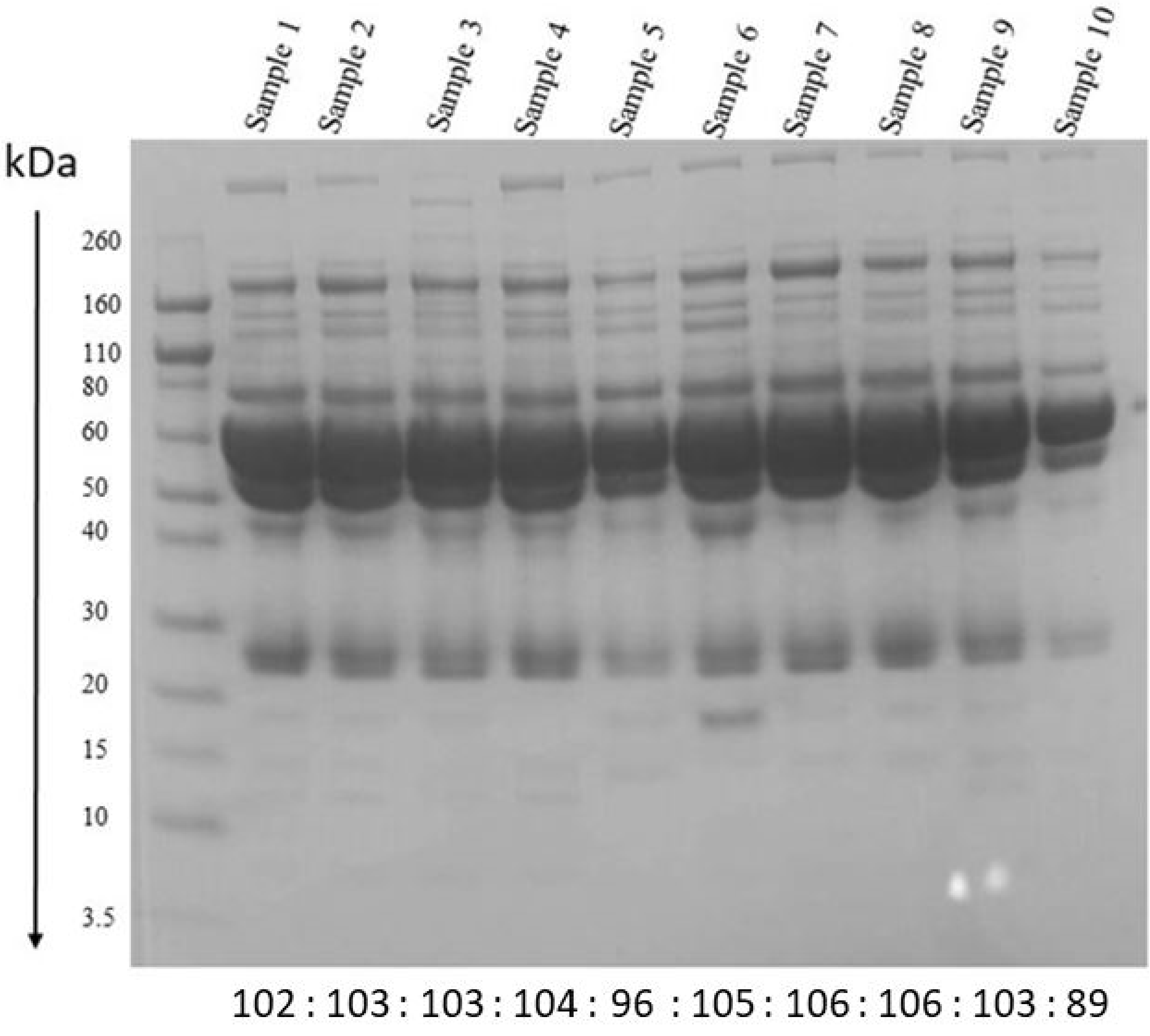


**Figure S1.** Serum samples from study subjects loaded on SDS-PAGE Gel to check the presence of proteins and get a rough idea of protein integrity: Lane 1: Ladder, Lane 2-6: Double positive RA patients for RF factor and anti-CCP, Lane 7-11: Single positive RA patients for RF factor. The integrated density ratio is shown at the bottom for each band. Integrated density ratio is calculated using ImageJ.

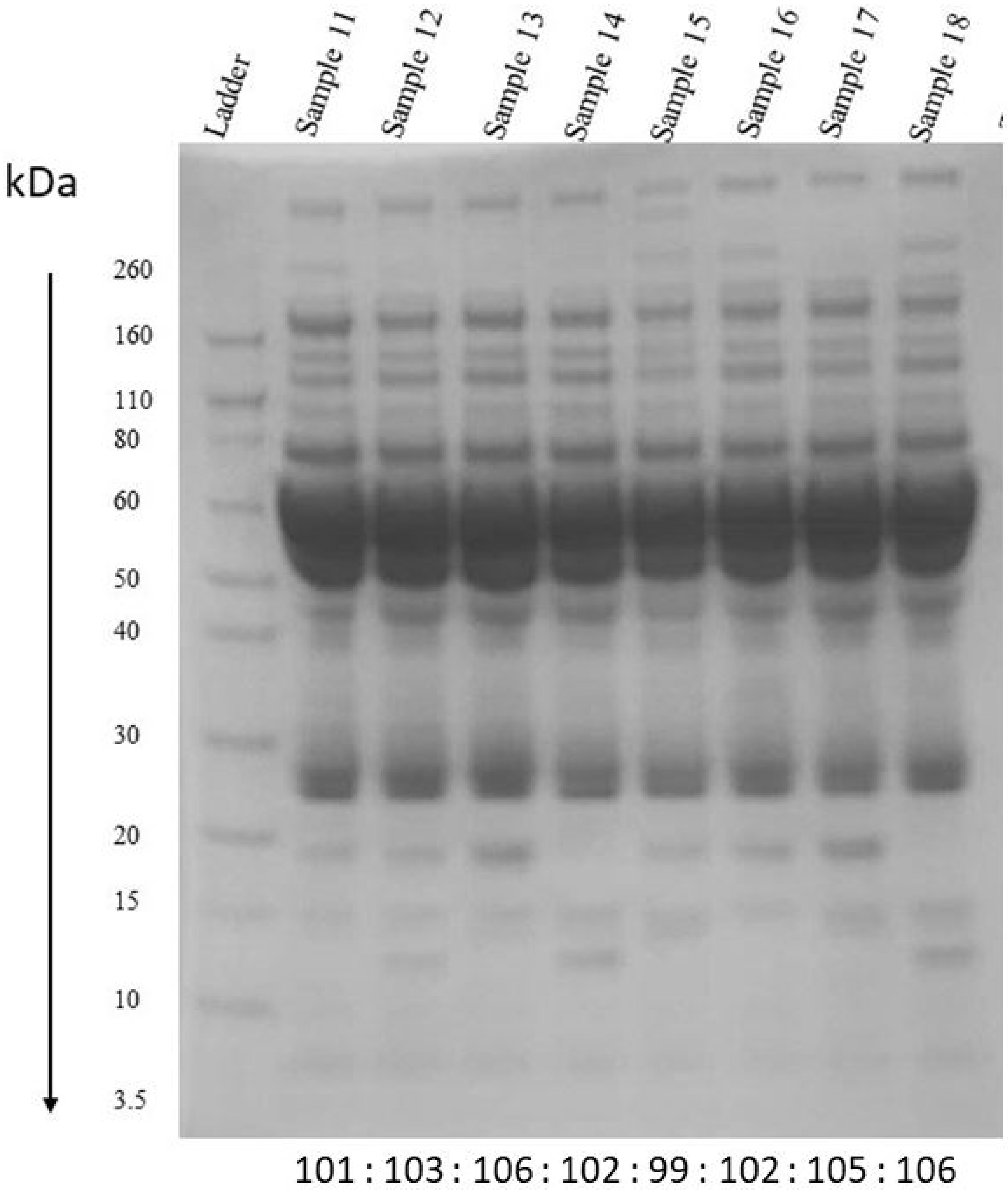


**Figure S2.** Serum samples from study subjects loaded on SDS-PAGE Gel to check the presence of proteins and get a rough idea of protein integrity: Lane 1: Ladder, Lane 2-6: Single positive RA patients for anti-CCP, Lane 7-9: Double negative RA patients for RF factor and anti-CCP. The integrated density ratio is shown at the bottom for each band. Integrated density ratio is calculated using ImageJ.

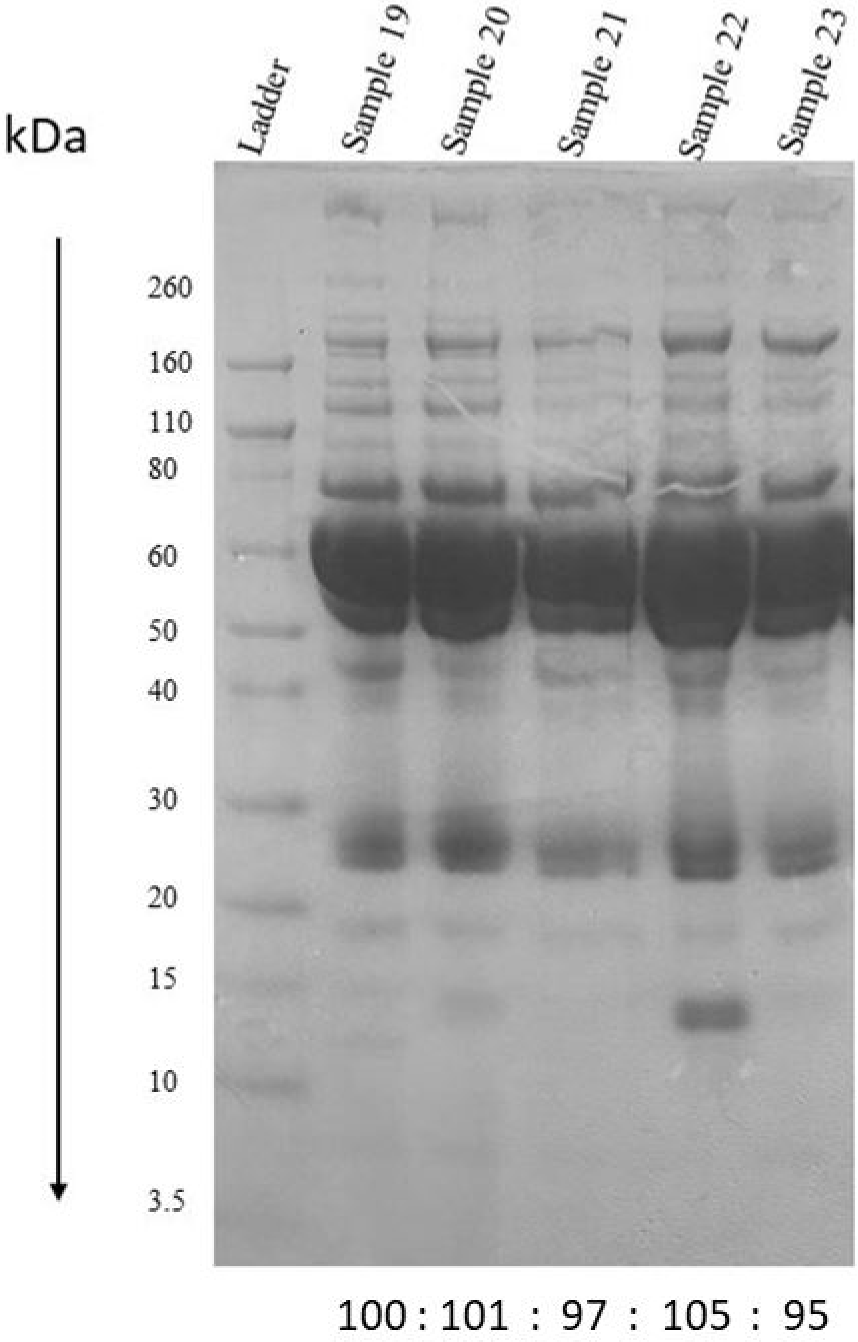


**Figure S3.** Serum samples from study subjects loaded on SDS-PAGE Gel to check the presence of proteins and get a rough idea of protein integrity: Lane 1: Ladder, Lane 2-8: Healthy control samples. The integrated density ratio is shown at the bottom for each band. Integrated density ratio is calculated using ImageJ.
